# Validation of qPCR reference genes in lymphocytes from patients with amyotrophic lateral sclerosis

**DOI:** 10.1371/journal.pone.0174317

**Published:** 2017-03-22

**Authors:** Ewa Usarek, Anna Barańczyk-Kuźma, Beata Kaźmierczak, Beata Gajewska, Magdalena Kuźma-Kozakiewicz

**Affiliations:** 1 Department of Biochemistry, Medical University of Warsaw, Warsaw, Poland; 2 Neurodegenerative Diseases Research Group, Medical University of Warsaw, Warsaw, Poland; 3 Department of Neurology, Medical University of Warsaw, Warsaw, Poland; Centre of Genomic & Post Genomics, ITALY

## Abstract

Quantitative polymerase chain reaction (qPCR) is the most specific and reliable method for determination of mRNA gene expression. Crucial point for its accurate normalization is the choice of appropriate internal control genes (ICGs). In the present work we determined and compare the expression of eight commonly used ICGs in lymphocytes from 26 patients with amyotrophic lateral sclerosis (ALS) and 30 control subjects. Peripheral blood mononuclear cells (PBMCs) before and after immortalization by EBV transfection (lymphoblast cell lines—LCLs) were used for qPCR analysis. LCLs were studied before and after liquid nitrogen cryopreservation and culturing (groups LCL1 and LCL2, respectively). qPCR data of 8 ICGs expression was analyzed by BestKeeper, NormFinder and geNorm methods. All studied genes (*18SRNA*, *ACTB*, *B2M*, *GUSB*,*GAPDH*, *HPRT1*, *MT-ATP6* and *RPS17*) were expressed in PBMCs, whereas only first four in LCLs. LCLs cryopreservation had no effect on ICGs expression. Comprehensive ranking indicated *RPS17* with *MT-ATP6* as the best ICGs for qPCR in PBMCs of control and ALS subjects, and *RPS17* with *18RNA* or *MT-ATP6* in LCLs from ALS. In PBMCs *18RNA* shouldn’t be used as ICG.

## Introduction

Amyotrophic lateral sclerosis (ALS) is a rare, incurable and fatal neurodegenerative disease characterized by progressive degeneration and loss of motor neurons, causing skeletal muscle weakness, wasting and death within 3–5 years from the first symptom onset [1]. The number of cases newly diagnosed with ALS each year is 1–3 per 100,000. About 90% of all diagnosed cases are sporadic (SALS) mostly of unknown origin, and the remaining 10% of individuals, with at least one other family member affected, have familial (FALS) [2]. Definitive diagnosis is difficult in early stages of ALS and its confirmation requires a period of observation [3]. There is no single, specific biochemical marker of ALS. Genetic testing may be helpful in diagnosis of FALS and its discrimination from SALS. Peripheral blood mononuclear cells (PBMCs) and human immortalized lymphoblast cell lines (LCLs) derived from PBMCs are a good and long-lasting source of nucleic acids for this type of studies.

At present, quantitative polymerase chain reaction (qPCR) is the most sensitive, specific, reliable and quick method for determination of mRNA gene expression [4]. However, alternations in the amount of starting material, RNA extraction, efficiency of reverse transcription and amplification may result in quantification errors [5]. Hence, the most important point for accurate normalization is the choice of appropriate internal control genes (ICGs) [6]. Initially, the housekeeping genes, the expression of which was supposed to be constant in different conditions, were used as ICGs. There are studies indicating that the mRNA expression of housekeeping genes may undergo regulation and significant changes during cell differentiation, pathological processes (malignancy, hypoxia) and may vary between patients [7–10].

Among the most commonly ICGs used for normalization in qPCR experiments are *18SRNA*, beta-actin (*ACTB*), beta-2-microglobulin (*B2M*), beta-glucuronidase (*GUSB*), glyceraldehyde-3-phosphate dehydrogenase (*GAPDH*), hypoxanthine phosphoribosyl transferase 1 (*HPRT1*), ATP synthase subunit 6 (*MT-ATP6*) and ribosomal protein S17 (*RPS17*) [4, 11–24]. They are frequently used without a proper validation of their expression stability. Many studies reported that the use of unstable ICGs leads to incorrect results [25–28].

To date, there are no studies concerning validation of ICGs for qPCR analysis in human lymphoblast cell lines. Thus, the aim of the present work was to determine and compare the expression of eight commonly used ICGs in PBMCs and LCLs (before and after liquid nitrogen storage) obtained from ALS patients.

## Materials and methods

### Patients and control subjects

The studies were conducted on peripheral blood mononuclear cells (PBMC) from 26 ALS patients diagnosed at the Department of Neurology, Medical University of Warsaw (13 women and 13 men), and from 30 age- and gender-matched control individuals without neoplastic and neurodegenerative disorders (14 women and 16 men). The mean age was 57.3 ± 14.5 for the patients and 57.3 ± 5.8 for control subjects. The controls were enrolled during a regular check up visit at their general practitioner.

Both the study protocol and the consent procedure were approved by the Bioethics Committee of the Medical University of Warsaw. Prior to enrollment to the study, each participant was given detailed “Information for the study participant” and signed an informed consent in 2 copies, one for each party.

### Separation of peripheral blood mononuclear cells

Venous blood samples from ALS patients and controls were collected in EDTA tubes and separated using a density gradient with Gradisol (AquaMed Poland). PBMCs were washed three times in PBS and used either for direct RNA isolation or for EBV transfection and a subsequent generation of immortalized primary lymphoblast cell lines (LCLs).

### Cell culture

LCLs were cultured at 37°C in a 5% CO_2_ atmosphere using RPMI-1640 medium (Invitrogen) supplemented with 20% FBS (Pan-Biotech), and antibiotics (pencillin 1%, streptomycin 1%, Sigma-Aldrich). On the fourth day, cyclosporine A (Sandimmun, Sandoz) was added to a final concentration of 1 μg/ml. The medium was changed twice a week for 4–6 weeks until the culture density reached 1–2 x 10^6^ cells/ml. The cell samples were divided and used either for RNA isolation (group LCLs1) or for liquid nitrogen cryopreservation in Bambanker freezing medium (Nippon Genetics) (group LCLs2). After at least 2 weeks in liquid nitrogen, the group LCLs2 was quickly thawed in 37°C water bath, washed with RPMI medium with 20% FBS and reconstituted by seeding on RPMI with 20% FBS. The cells were then cultured up to 1–2 x 10^6^ density and used for RNA isolation.

#### RNA isolation and qPCR

The total RNA was isolated from fresh PBMC using TriReagent solution (Ambion) according to manufacturer’s instructions. LCLs were suspended in TriReagent and homogenized in FastPrep-24 instrument (MP Biomedicals) using Lysing Matrix C (MP Biomedicals). To improve the isolation efficacy, 3 μl of Precipitation Carrier (MRC Inc) was added to each sample prior to isopropanol precipitation.

The total mRNA concentration was measured at 260 nm, and the purity assessed from absorbance ratio 260 nm/280 nm. Two μg of total RNA was reverse transcribed to a single-stranded cDNA according to the manufacturer’s instructions (Invitrogen, USA). Quantitative real-time PCR was performed in the StepOnePlus instrument (Applied Biosystems) using 400 ng of cDNA and a custom TaqMan® Array Plate. Internal control assays are listed in Table 1.

**Table 1:** Internal control assays on TaqMan® Array Plate.

Gene name	Gene symbol	Assay ID
18S ribosomal RNA	*18SRNA*	Hs99999901_s1
Actin, beta	*ACTB*	Hs99999903_m1
Beta-2-microglobulin	*B2M*	Hs99999907_m1
Glucuronidase, beta	*GUSB*	Hs99999908_m1
Glyceraldehyde-3-phosphate dehydrogenase	*GAPDH*	Hs99999905_m1
Hypoxanthine phosphoribosyltransferase 1	*HPRT1*	Hs99999909_m1
Mitchondrially encoded ATP synthase 6	*MT-ATP6*	Hs02596862_g1
Ribosomal protein S17	*RPS17*	Hs00734303_g1

#### Statistically-based methods

The validation of expression stability was calculated for each gene using Ct values and BestKeeper, NormFinder and geNorm statistical methods [29–31].

BestKeeper algorithm uses raw Ct values and calculates the variations (SD and CV). Unstable genes show SD > 1 and are considered unacceptable for further calculations. Determination of the most stable genes is based on the correlation coefficient (r) of their expression to the BestKeeper Index, which is the geometric mean of Ct values of the highly correlated candidate reference genes.

NormFinder is a model-based approach and works on both inter- and intragroup variations in gene expression, which are combined into a stability (S) value calculated for each single gene independently. The most stable candidate genes are characterized by the lowest S value.

GeNorm algorithm is based on the assumption that the expression ratio of the best two genes is stable in all studied samples. The software defines stability value (M), which is described as an average pair-wise variation of a gene of interest with all other tested genes. The gene with the lowest stability value has the most stable expression. GeNorm default threshold for stability value is 1.5.

Comprehensive ranking was calculated for individual ICGs as the geometric mean based on their weight according to all statistical methods used.

## Results

**Fig 1:**
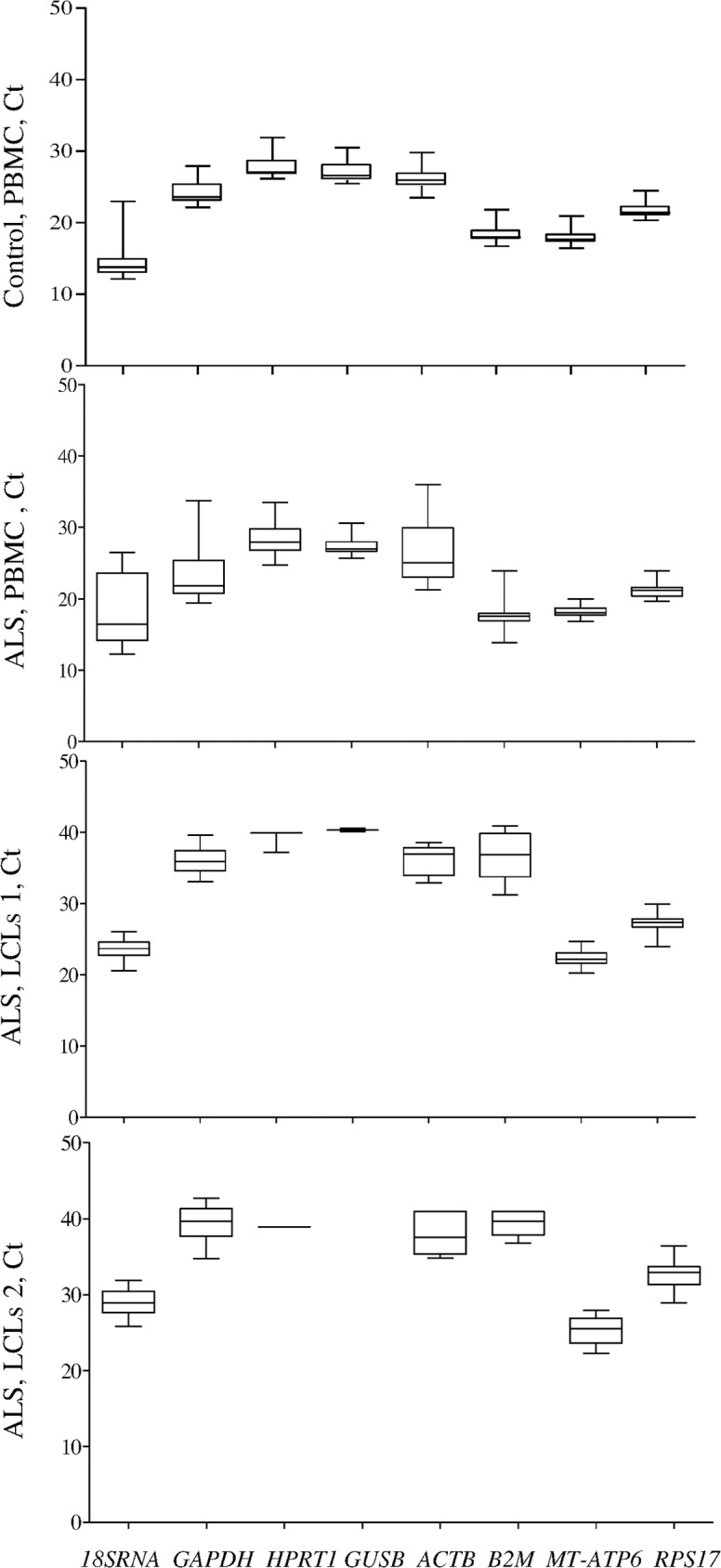
Ct values for candidate internal control genes (ICGs). The expression was studied as described in Material and Method section and expressed as medians (25th-75th percentile).

All studied genes were expressed in PBMCs of control and ALS cases and used for validation. The mean Ct value of studied genes is shown in Fig 1. It was similar in control and ALS cases ranging from 12 to 31 and from 12 to 35, respectively (Fig 1A and 1B).

In LCLs1 and LCLs2 of ALS patients the expression of only for four genes (*GAPDH*, *18SRNA*, *MT-ATP6* and *RPS17*) was detectable (Fig 1C and 1D). In LCLs1 the expression of *HPRT1*, *ACTB* and *B2M* was very low and detectable only in 3, 8, and 13 samples out of 26, respectively. In LCLs2 *HPRT1* was present only in 1, *ACTB* in 4 and *B2M* in 3 samples. *GUSB* expression was undetectable in neither LCL. The mean Ct value was higher in LCLs compared to PBMCs but similar in both groups (20–39 for LCLs1, 22–42 for LCLs2). LCLs cryopreservation had no effect on ICGs expression (Fig 1).

### BestKeeper analysis

In both the control and ALS PBMCs, there were three stable genes (SD < 1): *RPS17*, *MT-ATP6*, *B2M* and *RPS17*, *MT-ATP6*, *GUSB*, respectively. The strongest correlation was found for *RPS17 and MT-ATP6* in both groups, while in controls additionally for *B2M* (r > 0.9, p < 0.005) (Tables 2 and 3).

**Table 2:** Validation of internal control genes in PBMCs from control subjects.

Gene	Stability value				Comprehensive ranking
	BestKeeper		NormFinder	GeNorm	
	SD	r	ρ	M	
*RPS17*	0.75	0.98	0.455	0.346	1.26
*MT-ATP6*	0.73	0.96	0.565	0.346	1.71
*B2M*	0.97	0.97	0.532	0.485	2.62
*GUSB*	1.13	n/c	0.541	0.578	3.91
*HPRT1*	1.22	n/c	0.554	0.556	4.58
*GAPDH*	1.39	n/c	0.727	0.613	6.32
*ACTB*	1.13	n/c	1.023	0.840	5.81
*18SRNA*	1.61	n/c	2.336	1.239	8.00

n/c–not calculated

**Table 3:** Validation of internal control genes in PBMCs from ALS patients.

Gene	Stability value				Comprehensive ranking
	BestKeeper		NormFinder	GeNorm	
	SD	r	ρ	M	
*MT-ATP6*	0.58	0.91	1.542	0.580	1.19
*RPS17*	0.70	0.93	1.817	0.580	1.86
*HPRT1*	1.81	n/c	1.331	1.876	2.94
*GUSB*	0.95	0.88	2.202	0.783	3.46
*B2M*	1.24	n/c	2.806	1.318	4.68
*GAPDH*	3.03	n/c	2.711	2.528	5.73
*ACTB*	3.63	n/c	3.054	2.971	7.00
*18SRNA*	4.50	n/c	3.901	3.323	8.00

n/c–not calculated

Only *RPS17* and *MT-ATP6* exhibited SD < 1.0 in LCLs1 but the correlation (see chapter *Statistically-based methods*) was weaker but still strong (r > 0.6, p < 0.001) (Table 4). In LCLs2 for all genes were unstable (SD > 1.0), thus the correlation was not calculated (Table 5.).

**Table 4:** Validation of internal control genes in LCLs1 from ALS patients.

Gene	Stability value				Comprehensive ranking
	BestKeeper		NormFinder	GeNorm	
	SD	r	ρ	M	
*RPS17*	.83	0.66	0.678	0.938	1.26
*18SRNA*	.09	n/c	0.613	0.938	1.44
*MT-ATP6*	.83	0.69	1.046	1.126	2.08
*GAPDH*	.44	n/c	1.328	1.336	4.00

n/c–not calculated

**Table 5:** Validation of internal control genes in LCLs2 from ALS patients.

Gene	Stability value				Comprehensive ranking
	BestKeeper		NormFinder	GeNorm	
	SD	r	ρ	M	
*18SRNA*	1.24	n/c	0.302	0.348	1.26
*RPS17*	1.28	n/c	0.174	0.348	1.44
*MT-ATP6*	1.26	n/c	0.400	0.398	2.62
*GAPDH*	1.90	n/c	0.914	0.677	4.00

n/c–not calculated

### NormFinder analysis

The most stable genes in control PBMCs were *RPS17* and *B2M* while in ALS—*MT-ATP6* and *HPRT1* (ρ < 1). The expression of *18SRNA* was the most variable (ρ > 3) (Tables 2 and 3). In contrast, in LCLs1 and LCLs2 the *18SRNA* was ranked on the first and the second position (respectively) as compared to other studied genes. In both LCLs the *RPS17* and *MT-ATP6* were also stable, whereas *GAPDH* was unstable (last position) (Tables 4 and 5).

### GeNorm analysis

All studied genes were stable (M < 1.5) in PBMCs of control samples, with the best being *RPS17* and *MT-ATP6*. In ALS cases only four genes were stable, with the best results of *MT-ATP6* and *RPS17*, too (Tables 2 and 3). In both PBMCs the *18SRNA* showed the lowest stability. In LCLs1 and LCLs2 all genes were stable. The *18SRNA* showed better stability in LCLs compared to PBMCs (Tables 4 and 5).

## Discussion

Real-time PCR has become a standard method for quantification of gene expression in different experimental conditions. For reliable and accurate analysis of qPCR data, it is critical to select proper ICGs able to eliminate non-biological variations [6, 32]. There are no universal ICGs, the expression of which is stable among different tissues and various conditions. Thus, identification and validation of control genes is an absolutely essential step before experimental settings [33]. The guidelines of MIQE (Minimum Information for Publication of Quantitative Real-Time PCR Experiments), recommend normalization against more than one reference gene at a time [34].

Despite a high number of studies, the molecular mechanisms of sporadic ALS remain unclear and a specific disease marker is still lacking [35, 36]. Restricted access to human nervous tissue has promoted the use of transgenic animals with ALS-like symptoms [37–39]. Although very useful, they do not always reflect the clinical and pathological changes present in human subjects. Studies on biological markers are therefore conducted in human CSF and blood serum [40, 41]. Also the lymphocytes are an easily accessible source of material both for genetic and molecular studies. Moreover, establishment of LCLs (by EBV transfection) potentially provides an unlimited source of biologic material. The LCLs can be either cultured and used for immediate studies, or frozen and preserved for future experiments. However, it is not known whether the procedures, which lead to generation of LCLs from PBMCs, have any influence on the expression of commonly used ICGs, and—as a consequence—on the final results obtained by qPCR.

Although there is a number of papers concerning the validation of ICG in lymphocytes [42, 43], there are no reports addressing this issue in LCLs. Moreover, to our knowledge, there have been no studies comparing the stability of ICG between the fresh PBMCs and LCLs obtained from the same individuals.

In the present study we evaluated eight commonly used ICGs for their usefulness in gene expression analysis by qPCR in PBMCs and LCLs before and after freezing. All studied genes (*18SRNA*, *ACTB*, *B2M*, *GUSB*, *GAPDH*, *HPRT1*, *MT-ATP6* and *RPS17*) were expressed in PBMCs, whereas only the first four in LCLs. LCLs cryopreservation had no effect on ICGs expression. According to the comprehensive ranking, the expression of *RPS17* and *MT-ATP* was the most stable in PBMCs of both ALS and control subjects. The most variable genes were *ACTB* and *18SRNA*, which had previously been used as ICGs in studies performed in PBMCs of patients with ALS and other diseases [12–15]. Our result on the variability of *ACTB* are in agreement with the latest studies of Zhang et al. [19] who classified them as unstable in PBMC of patients with chronic hepatitis B. According to Zhang, the best pair of ICGs for their studies were *GAPDH* and *beta-tubulin*, however in our ranking the *GAPDH* was localized on the sixth position in both ALS and control PBMCs. Barber et al. [44], who studied *GAPDH* as a housekeeping gene in 72 human tissues, showed a 15-fold difference in its expression. As in our PBMCs, the expression of *ACT*, *18SRNA*, and *GAPDH* was also found to vary considerably in asthmatic airways [20].

In LCL1 and LCL2 (before and after liquid nitrogen cryopreservation), unlike in the PBMCs, the expression of *HPRT1*, *ACTB*, *B2M*, and *GUSB* was very low or undetectable. Even though *B2M* was used as ICGS in some previous studies [16, 17, 45], we found it unsuitable for qPCR normalization in LCLs. Also the *GAPDH*, which appeared unstable in our LCLs, served as the ICG earlier [45]. Our study showed that due to a high stability, the *18SRNA*, *RPS17*, and *MT-ATP6* are good reference genes for qPCR studies in LCLs. The *18SRNA*, stable in LCLs, should not however be considered in studies performed simultaneously in PBMCs and LCLs.

Interestingly, we found the mitochondrial gene *MT-ATP6*, rarely used as ICG, to be very stable in PBMCs. It was ranked on the first position in ALS, the second in the controls, and the third in both LCLs. *MT-ATP6* was also one of the most stably-expressed control genes among 32 studied by Jones et al. in eight human tissues [21]. The *RPS17*, earlier described among the most stably expressed genes in carcinoma cells [23], was highly stable in control PBMCs and LCL1 (the first position in the comprehensive ranking), as well as in PBMCs of ALS and LCL2 (the second position).

To sum up, we recommend using *MT-ATP6* and *RPS17* as the best pair of reference genes for expression in PBMCs, *18SRNA* and *RPS17* in LCLs, whereas *RPS17* and *MT-ATP6* in studies concomitantly performed in PBMCs and LCLs.

## Supporting information

S1 Table Mean Ct values for reference genes candidates in control and ALS PBMCs (XLS)

S2 Table Mean Ct values for reference genes candidates in LCLs (XLS)
